# Effects of low-carbohydrate, high-nutrient-density diet combined with aerobic exercise on mental health and glycolipid metabolism in obese children and adolescents

**DOI:** 10.3389/fmed.2026.1745779

**Published:** 2026-01-30

**Authors:** Long Li, Peiwen Zhang, Xingyu Liu, Chun Wang

**Affiliations:** 1 School of Sports Medicine and Health, Chengdu Sport University, Chengdu, Sichuan, China; 2 College of Physical Education, Xichang University, Xichang, Sichuan, China; 3 College of Nursing and Rehabilitation, Xi’an Fanyi University, X’an, Shaanxi, China; 4 School of Biology and Food Engineering, Changshu Institute of Technology, Suzhou, Jiangsu, China

**Keywords:** aerobic exercise, children and adolescents, glycolipid metabolism, low-carbohydrate and high-nutrient-density diet, mental health, obese

## Abstract

**Objective:**

To explore the effects of low-carbohydrate, high-nutrient-density diet combined with aerobic exercise on mental health and glycolipid metabolism in children and adolescents with obesity.

**Methods:**

A total of 121 children and adolescents with obesity treated in our hospital from January 2022 to December 2023 were enrolled in this study and divided into a control group (*n* = 60) and a study group (*n* = 61). The control group received balanced diet, lifestyle, and exercise education. The study group adopted a low-carbohydrate and high-nutrient-density diet, restricting carbohydrate intake while emphasizing foods rich in essential nutrients like vitamins and minerals, combined with daily aerobic exercise (swimming, jogging, skipping, and badminton, running, or climbing stairs, 30–60 min per session). The intervention lasted for 8 weeks. The body fat metabolism, blood lipid parameters, blood glucose related indexes, psychological status, and quality of life, were compared in the two groups.

**Results:**

After intervention, body mass index (BMI), body fat content, body fat percentage, and waist-to-hip ratio were lower in the study group [(17.56 ± 1.72) kg/m^2^, (17.41 ± 1.75) kg, (38.01 ± 3.82) %, and (0.72 ± 0.07)] compared with the control group [(19.32 ± 2.01) kg/m^2^, (19.02 ± 1.95) kg, (41.78 ± 4.18) %, and (0.89 ± 0.09)] (*p* < 0.05). After intervention, the study group exhibited lower improvements in total cholesterol (TC) and low-density lipoprotein (LDL-C) and more favorable triglyceride (TG) and high-density lipoprotein (HDL-C) [(4.06 ± 0.41) mmol/L, (4.26 ± 0.43) mmol/L, (1.16 ± 0.12) mmol/L, and (1.65 ± 0.17) mmol/L] compared with the control group [(5.91 ± 0.60) mmol/L, (3.65 ± 0.36) mmol/L, (1.62 ± 0.16) mmol/L, and (1.38 ± 0.14) mmol/L] (*p* < 0.05). After intervention, the levels of fasting blood glucose (FPG), C-peptide (C-P), fasting insulin (FINS), and homeostasis model islet resistance index (HOMA-IR) were lower in the study group [(4.82 ± 0.48) mmol/L, (5.18 ± 0.52) μU/mL, (539.65 ± 53.24) pmol/L, and (1.12 ± 0.11)] when compared with the control group [(5.45 ± 0.55) mmol/L, (8.62 ± 0.86) μU/mL, (723.15 ± 72.35) pmol/L, and (1.92 ± 0.19)] (*p* < 0.05). After intervention, the self-rating anxiety scale (SAS) and self-rating depression scale (SDS) scores were lower in the study group [(36.28 ± 3.62) points and (37.26 ± 2.71) points] when compared with the control group [(42.23 ± 4.23) points and (43.82 ± 4.38) points] (*p* < 0.05). After intervention, the scores of social, psychological, physical, and emotional function were higher in the study group [(86.65 ± 8.21) points, (83.54 ± 8.35) points, (85.62 ± 8.46) points, and (86.21 ± 8.61) points] than those in the control group [(80.23 ± 8.01) points, (78.21 ± 7.82) points, (80.32 ± 8.14) points, and (80.15 ± 8.06) points] (*p* < 0.05).

**Conclusion:**

Low-carbohydrate and high-nutrient-density diet combined with aerobic exercise significantly improves mental health and glycolipid metabolism in children and adolescents with obesity.

## 1 Introduction

With the improvement of socio-economic level and the change in life concepts, the obesity rate among teenagers is rising steadily, exerting a significant impact on their health (1). Globally, there are about 1.5 billion overweight or obese individuals and 31% of children and adolescents fall in this category (2). According to China’s 2019 data, the total obesity rate among children and adolescents reached 19%, with the incidence rate showed an increasing trend annually (3).

On the one hand, obesity in children and adolescents is influenced by genetic factors (4). On the other hand, poor eating habits and insufficient exercise further promote the occurrence and development of obesity (5). Research suggests that obesity in childhood and adolescence elevates the risk of obesity in adulthood. Specifically, 40% of children with obesity become obese adolescents, and 75–80% of obese adolescents remain obese as adults (6). Obesity not only affects the health of children and adolescents, causing long-term chronic damage to cardiovascular and respiratory functions, but also increases the risk of hypertension, diabetes, coronary heart disease, and other related diseases, and is a main risk factor for metabolic syndrome (7).

In addition to the well-known impacts on physical health, obesity in children and adolescents also has significant negative effects on their mental health (8). Children and adolescents with obesity are more likely to experience body image dissatisfaction, low self-esteem, and social isolation, and are at a higher risk of developing mental health disorders such as anxiety and depression (9). Mental and physical health are closely interrelated, poor mental health can further exacerbate physical health problems, creating a vicious cycle (10). Therefore, improving mental health in children and adolescents with obesity is critical for their overall well-being and quality of life.

In the 1960s, American doctor Cooper proposed the term “aerobic exercise” (11). In his book *Aerobic Exercise Metabolism*, he defined aerobic exercise as a form of physical activity performed with adequate oxygen supply, enabling the body to obtain sufficient energy through aerobic metabolic reactions (12). In our study, we selected several types of aerobic exercises that are well-suited for children and adolescents, considering their physical fitness levels and sports preferences. These exercises include swimming, jogging, skipping rope, playing badminton, running, and climbing stairs. Swimming provides a full-body exercise that enhances cardiovascular endurance and muscle strength while minimizing joint stress (13). Jogging and running are simple yet effective methods to improve aerobic capacity and burn calories (14). Skipping rope is a convenient and high-intensity aerobic activity that can be easily incorporated into daily routines (15). Badminton combines running, jumping, and rapid movements, improving agility and coordination while providing an aerobic workout (16). Climbing stairs is a practical exercise that can be done at home or in public places, effectively increasing heart rate and promoting fat burning (17).

Dietary intervention is also a safe and effective way to lose weight in both children and adolescents (18). Energy-limited starvation therapy and fasting patterns are effective for weight loss, but they are difficult to implement and adhere to in children and adolescents (19). There is an urgent need to find an effective, hunger-free, high-nutrition, easy-to-implement, and easy-to-adhere-to weight-loss dietary pattern to prevent and control childhood and adolescent obesity. This has far-reaching significance for solving the increasingly severe national obesity problem (20).

A low-carbohydrate and high-nutrient-density diet is a dietary approach that restricts carbohydrate intake while emphasizing the consumption of foods rich in essential nutrients such as vitamins, minerals, and dietary fiber (21). In this dietary pattern, the daily carbohydrate intake is typically limited to below 100–150 g per day, depending on individual energy requirements and metabolic status (22). High-nutrient-density foods include vegetables, fruits, lean proteins (such as fish, poultry, and legumes), and healthy fats (such as nuts and seeds). This dietary approach aims to provide adequate nutrients while promoting fat metabolism and weight control by reducing the reliance on carbohydrates as the primary energy source (23). Previous studies have explored the association between low-carbohydrate and high-nutrient-density diets and obesity, as well as underlying mechanisms. In adults, low-carbohydrate and high-nutrient-density diets have been shown to be effective in reducing weight, and have been included in the American Cardiovascular Society Guidelines for weight loss (24). Mechanistically, reducing carbohydrate intake lowers insulin levels. Lower insulin levels facilitate the breakdown of stored fat, enabling the body to use fat as a primary energy source instead of relying on carbohydrates (a process known as ketosis in more carbohydrate-restricted diets). This shift in energy metabolism can contributes to weight loss and improvements in body composition (25).

In children and adolescents, although few studies compared with adults, preliminary evidence showing promising results. For instance, a study indicated that low-carbohydrate and high-nutrient-density diets improved body composition and metabolic parameters in this population (26). However, the effectiveness and safety of low-carbohydrate and high-nutrient-density diets in children and adolescents still need further evidence, especially considering their unique growth and development needs.

The novelty of our study lies in the following three key aspects. Firstly, we comprehensively combined a low-carbohydrate and high-nutrient-density diet with a set of aerobic exercises specifically selected for children and adolescents with obesity, considering their unique physical and psychological characteristics. Secondly, we not only focused on the traditional outcomes, such as weight loss and body composition changes, but also simultaneously evaluated the effects on mental health and glycolipid metabolism, to provide a more holistic view of the intervention’s impact on this vulnerable population. Thirdly, we developed a personalized low-carbohydrate and high-nutrient- density diet plan based on the individual growth and development needs of children and adolescents, an approach that has not been extensively explored in previous studies.

This study aimed to explore the effects of low-carbohydrate and high-nutrient-density diet combined with aerobic exercise on mental health and glycolipid metabolism in children and adolescents with obesity.

## 2 Data and methods

### 2.1 General data

A total of 121 children and adolescents with obesity treated in our hospital from January 2022 to December 2023 were chosen to be the study participants. They were divided into control group (*n* = 60) and study group (*n* = 61), based on the random number table method.

Inclusion criteria: (1) age 7 ~ 18 years old; (2) body mass index (BMI) met the obesity standards for children and adolescents; (3) patients were mentally and cognitively normal and can complete the investigation with parents cooperation; and (4) patient and guardian agreed and signed the informed consent.

Exclusion criteria: (1) obesity due to disease (pathological obesity) or drug-related obesity; (2) combined with moderate or severe hepatic and renal insufficiency; (3) patients with acute, chronic diseases and cardiopulmonary dysfunction; and (4) patients with congenital body disorders.

## 3 Methods

The control group received comprehensive interventions encompassing balanced diet, lifestyle modification, and exercise education.

The balanced diet education was delivered through a series of structured online sessions designed to provide in-depth knowledge about the balanced diet principles. The key components included an appropriate macronutrient distribution including: 50–65% carbohydrates (mainly sourced from whole grains, vegetables, and fruits), 15–20% protein (lean meats (such as chicken and fish), legumes, and dairy products), and 20–30% fat (emphasizing healthy fats source like nuts, seeds, and olive oil).

In addition to macronutrient distribution, the education also covered portion control. Participants were taught how to estimate appropriate serving sizes using visual cues (e.g., serving of meat compared to the size of a deck of cards). Moreover, they were educated on the importance of consuming a variety of foods to ensure a sufficient intake of micronutrients, including vitamins and minerals. These online sessions were conducted weekly for 8 weeks, allowing participants to gradually absorb and apply the knowledge.

The lifestyle modification education was provided through group counseling sessions. These sessions were held bi-weekly in a community center, creating an interactive and supportive environment for the participants. The main topics covered included sleep hygiene, stress management, and smoking cessation (for participants who smoked).

For sleep hygiene, participants were educated on the importance of maintaining a regular sleep schedule, creating a comfortable sleep environment, and avoiding stimulants like caffeine before bedtime. Stress management techniques included deep breathing exercises, meditation, and progressive muscle relaxation. The group format allowed participants to share their experiences and learn from one another, enhancing the effectiveness of the education.

The exercise education was delivered as a combination of online courses and in-person demonstrations. Online courses provided theoretical knowledge about exercise types, benefits, and safety precautions. Participants learned about the importance of regular physical activity for weight management and overall health.

The in-person demonstrations were held monthly at a local gym. During these sessions, professional fitness trainers demonstrated proper exercise techniques for various activities, including aerobic exercises (such as brisk walking and cycling) and strength training exercises (e.g., dumbbells and resistance bands). Individualized exercise plans based on fitness levels and health conditions, with participants encouraged to gradually increase the intensity and duration.

Each patient in the control group was equipped with a body fat scale, and instructed to measure their weight and body fat daily, uploading the data to an associated APP. Nutritionists registered the changes in the patient’s weight and body fat daily, monitoring the progress and providing timely feedback and adjustments to the intervention plans as needed.

The study group adopted low-carbohydrate and high-nutrient-density diet combined with aerobic exercise.

For low-carbohydrate and high-nutrient-density diet: (1) dietitian first conducted a detailed assessment of the patients’ eating and living habits, and at the same time, the patients and their guardians received education on diet, lifestyle and exercise. (2) Considering that the total carbohydrate in the diet of Chinese children and adolescentsis relatively high, and referring to the proportion of low-carbohydrate diets in foreign studies, the nutrient distribution ratio of low carbon and high nutrient density diet in this study was set as follows: carbohydrate 20–30% (< 100 g/d), protein 40–50%, and fat 20–30%. Nutritionists calculated each patient’s total caloric requirements based on ideal body weight, body fat content, and daily physical activity intensity, thereby developing a personalized low carbon and high income diet. (3) To achieve a standardized diet, no hunger, and ease of operation and implementation at home, the patients in the study group adopted nutrition bars with low carbon, high protein, high fiber, and rich vitamins and minerals to replace staple foods (such as rice noodles for lunch and dinner), focusing on limiting refined rice noodles and high-starch, high-fat and sugar-containing foods, and eating vegetables, fruits, meat, eggs, milk, nuts, and soy products under the guidance of nutritionists on demand. (4) Patients took daily multivitamin tablets and calcium, magnesium, and zinc supplements, and drank plenty of water. To ensure the effective implementation of dietary intervention, full-time dietitians and health managers in obesity clinics followed up patients daily through Wechat to guide patients’ diet.

For aerobic exercise: Exercise sessions were organized under the supervision of professional fitness trainers. Before starting the exercise program, each child underwent a comprehensive physical fitness assessment, including tests for cardiovascular endurance, muscular strength, and flexibility. Based on the assessment results and the children’s sports hobbies, appropriate exercise methods were selected, including swimming, jogging, skipping, badminton, running, or stair climbing.

Each exercise session lasted 30–60 min, once daily. The trainers used heart rate monitors and perceived exertion scales to objectively measure and adjust the exercise intensity. The goal was to have the children reach an intensity where they felt mild fatigue, which was equivalent to a heart rate range of 60–80% of their maximum heart rate (calculated as 220 minus age).

As the children’s physical fitness improved, the trainers gradually increased the frequency and duration of exercise. They also provided real-time feedback and encouragement during the sessions to ensure safety and motivation. Additionally, the trainers recorded detailed exercise data for each child, including the type of exercise, duration, intensity, and any special circumstances, to monitor progress and make further adjustments as needed.

The intervention lasted for 8 weeks in both groups.

### 3.1 Observation indicators

All observation indicators were measured before and after 8 weeks of intervention.

(1) Body fat metabolism: BMI, body fat content, body fat percentage, and waist-to-hip ratio of children were compared between the two groups. BMI was calculated by dividing weight in kilograms by the square of height in meters (kg/m^2^). Body fat content and body fat percentage were measured using a bioelectrical impedance analysis (BIA) device (InBody 770 BIA analyzer). Waist-to-hip ratio was determined by dividing the waist circumference (measured at the narrowest point) by hip circumference.
(2) Blood lipid parameters: A total of 6 mL of fasting venous blood was collected from the elbow of each participant. The blood samples were placed in anticoagulant-free tubes and allowed to clot at room temperature for 30 min and subsequently centrifuged at 3000 rpm for 10 min to separate the serum. The serum levels of LDL-C, TC, TG, and HDL-C were then detected using an automatic biochemical analyzer (Beckman Coulter, United States).
(3) Blood glucose related indexes: A total of 6 mL of fasting venous blood was collected from the elbow of each participant. The blood samples were immediately placed in anticoagulant tubes containing ethylenediaminetetraacetic acid (EDTA) to prevent clotting and plasma was separated by centrifugation at 3000 rpm for 10 min. FPG levels were measured using a glucose oxidase method. C-P and FINS levels were measured using enzyme-linked immunosorbent assay (ELISA) kits. The specific product models used were as follows: C-P was measured using D711370-0048, provided by Sangon Biotech; FINS was measured using JLC6649-96 T, provided by Shanghai Jing Kang Biotechnology Co., Ltd. The homeostasis model islet resistance index (HOMA-IR) was calculated using the formula: HOMA-IR = (FINS×FPG)/22.5.
(4) Psychological status: SAS and SDS were assessed to evaluate the psychological status of patients (27). Higher scores indicated more serious anxiety and depression, with the threshold of ≥50 for anxiety and ≥53 for depression.
(5) Quality of life: Short Form 36 Health Survey (SF-36) was used to assess the social, psychological, physical, and emotional function of patients (28). The maximum score of each item was 100 points, and the score was proportional to the quality of life of patients.

### 3.2 Statistical analysis

SPSS 24.0 statistical software was implemented for data analysis. Measurement data were expressed as (x ± s), and repeated measurement analysis of variance (ANOVA) was used to compare the measurement data at multiple time points. Count data were expressed as (*n*, %), and *χ*^2^ test was used for comparison. 95% confidence intervals (CIs) were calculated as effect size. A value of *p* < 0.05 indicates that the difference is statistically significant.

## 4 Results

### 4.1 Baseline data between the two groups

The control group contained 30 female and 30 male patients, aged from 7 to 18 years old, with an average age of (12.35 ± 1.69) years. The study group contained 32 female and 29 male patients, aged from 8 to 18 years old, with an average age of (12.38 ± 1.72) years. There were no differences in general data between the two groups (*p* > 0.05, Table 1).

**Table 1:** Baseline data between the two groups.

Groups	Cases	Gender		Age (years)
		Male	Female	
Control group	60	30 (50.00)	30 (50.00)	12.35 ± 1.69
Study group	61	32 (52.46)	29 (47.54)	12.38 ± 1 0.72
*χ*^2^/*t*		0.073		0.096
*p*		0.786		0.923

### 4.2 Body fat metabolism between the two groups

**Figure 1:**
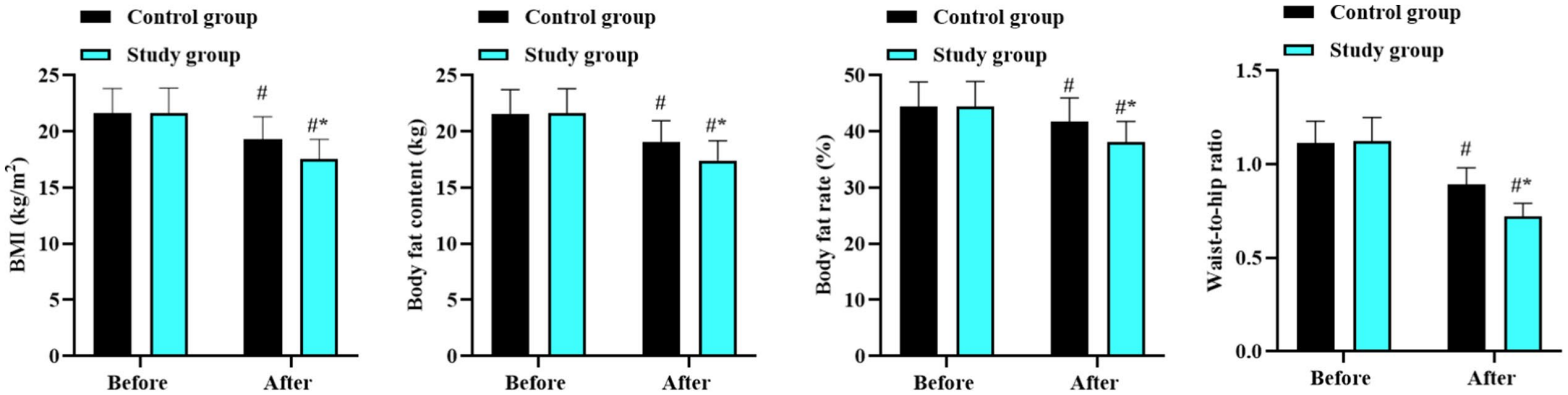
Body fat metabolism in the two groups. ^#^*p* < 0.05, in contrast to before intervention. ^*^p < 0.05, in contrast to control group.

Before intervention, there were no differences in BMI, body fat content, body fat percentage, and waist-to-hip ratio between the two groups (*p* > 0.05). After intervention, BMI, body fat content, body fat percentage, and waist-to-hip ratio reduced in the two groups (*p* < 0.05, 95% CI: 2.722–3.748; *p* < 0.05, 95% CI: 2.864–3.886; *p* < 0.05, 95% CI: 3.431–5.579; and *p* < 0.05, 95% CI: 0.283–0.336), and the ratios in the study group were lower than the control group (*p* < 0.05, 95% CI: 0.362–1.399; *p* < 0.05, 95% CI: 0.264–1.286; *p* < 0.05, 95% CI: 0.791–2.939; and *p* < 0.05, 95% CI: 0.053–0.1.06; Figure 1).

### 4.3 Blood lipid levels between the two groups

**Figure 2:**
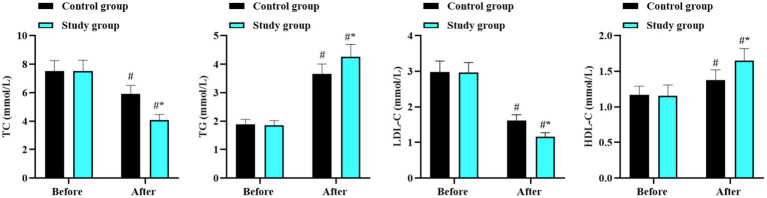
Blood lipid levels in the two groups. ^#^*p* < 0.05, in contrast to before intervention. ^*^p < 0.05, in contrast to control group.

Before intervention, there were no differences in the levels of TC, TG, LDL-C, and HDL-C between the two groups (*p* > 0.05). After intervention, the levels of TC and LDL-C reduced while the levels of TG and HDL-C increased in both groups (*p* < 0.05, 95% CI: 2.372–2.698; *p* < 0.05, 95% CI: −2.168 to −2.012; *p* < 0.05, 95% CI: 1.526–1.644; and *p* < 0.05, 95% CI: −0.38 to --0.313). Moreover, the improvements in these lipid indicators in the study group were better than those in the control group (*p* < 0.05, 95% CI: 0.751–1.078; *p* < 0.05, 95% CI: −0.367 to −0.213; *p* < 0.05, 95% CI: 0.176–0.293; and *p* < 0.05, 95% CI: −0.167 to −0.092; Figure 2).

### 4.4 Blood glucose related indexes between the two groups

**Figure 3:**
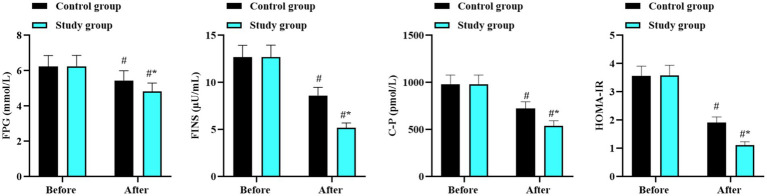
Blood glucose related indexes in the two groups. ^#^*p* < 0.05, in contrast to before intervention. ^*^p < 0.05, in contrast to control group.

Before intervention, there were no differences in the levels of FPG, FINS, C-P, and HOMA-IR between the two groups (*p* > 0.05). After intervention, the above blood glucose related indexes reduced in both groups (*p* < 0.05, 95% CI: 0.959–1.250; *p* < 0.05, 95% CI: 5.535–6.055; *p* < 0.05, 95% CI: 329.2–371.1; and *p* < 0.05, 95% CI: 1.981–2.119), and those in the study group were lower than those in the control group (*p* < 0.05, 95% CI: 0.159–0.450; *p* < 0.05, 95% CI: 1.445–1.965; *p* < 0.05, 95% CI: 70.77–112.7; and *p* < 0.05, 95% CI: 0.320–0.459; Figure 3).

### 4.5 Psychological status between the two groups

**Figure 4:**
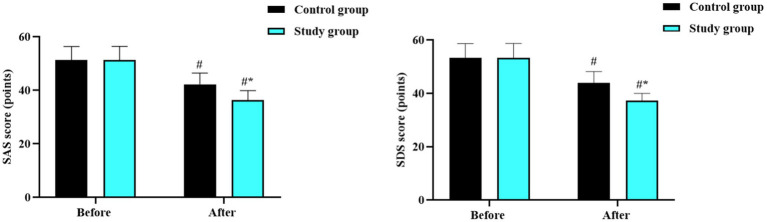
Psychological status in the two groups. ^#^*p* < 0.05, in contrast to before intervention. ^*^p < 0.05, in contrast to control group.

Before intervention, there were no differences in the SAS and SDS scores between the two groups (*p* > 0.05). After intervention, the SAS and SDS scores reduced in both groups (*p* < 0.05, 95% CI: 10.84–13.16; *p* < 0.05, 95% CI: 11.65–13.97), and those in the study group were lower than those in the control group (*p* < 0.05, 95% CI: 1.811–4.129; *p* < 0.05, 95% CI: 2.114–4.426; Figure 4).

### 4.6 Quality of life between the two groups

**Figure 5:**
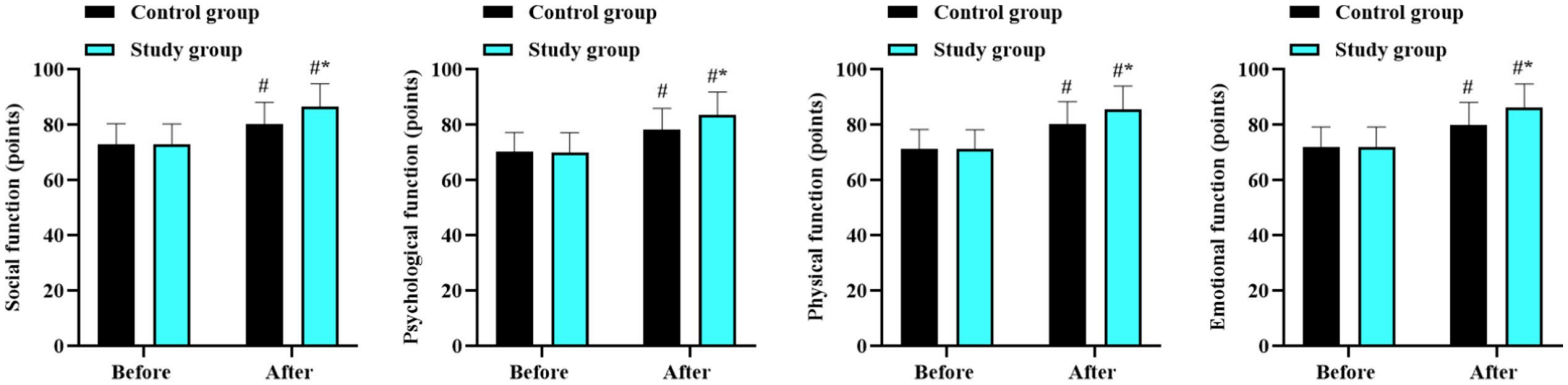
Quality of life in the two groups. ^#^*p* < 0.05, in contrast to before intervention. ^*^p < 0.05, in contrast to control group.

Before intervention, there were no differences in the scores of social, psychological, physical, and emotional function scores between the two groups (*p* > 0.05). After intervention, the scores in all domains improved significantly in both groups (*p* < 0.05, 95% CI: −12.26 to −8.351); *p* < 0.05, 95% CI: −12.60 to −8.762; and (*p* < 0.05, 95% CI: −13.68 to −9.768); (*p* < 0.05, 95% CI: −13.05 to −9.099). Improvements were higher in the study group compared with the control group (*p* < 0.05, 95% CI: −5.149 to −1.241; *p* < 0.05, 95% CI: −9.236 to −1.564; *p* < 0.05, 95% CI: −4.592 to −0.677; and *p* < 0.05, 95% CI: −4.991 to −1.039; Figure 5).

## 5 Discussion

Obesity in children and adolescents has emerged as a major public health issue, with central obesity being particularly prevalent and directly impacting overall metabolism, leading to a variety of metabolic diseases and imposing substantial economic and social burden (29). Dietary factors are the primary contributors to obesity in this population, making dietary intervention a safe and effective approach for weight loss (30). Among various dietary strategies, low-carbohydrate diets have shown promise, with studies indicating its superiority over a low-fat diet for weight loss (31). However, the specific application of low-carbohydrate diets in childhood and adolescent obesity, as well as the development of individualized low-carbohydrate, high-nutrient-density diets, requires further investigation.

The low-carbohydrate and high-nutrient-density plan formulated in this study accounted the unique growth and developmental needs of children. By setting the nutrient distribution ratio at 20–30% for carbohydrates (< 100 g/d), 40–50% for protein, and 20–30% for fat, and calculating the total caloric requirement for each patient based on their individual circumstances, to ensure a balanced and safe dietary intervention. Compared to the Mediterranean diet, which emphasizes whole grains, fruits, vegetables, legumes, nuts, and olive oil, our low-carbohydrate, high-nutrient-density diet focuses more on reducing carbohydrate intake while maintaining adequate protein and fat intake to support growth and development. Both diets have their merits, and the choice between them may depend on individual preferences, cultural background, and specific health goals.

Our study demonstrated that after the intervention, BMI, body fat content, body fat percentage, and waist-to-hip ratio decreased in both groups, with more significant improvements in the study group receiving the low-carbohydrate and high-nutrient-density diet combined with aerobic exercise. These findings suggest that this combined approach can effectively improve body fat metabolism in children and adolescents with obesity. The potential mechanisms include: (1) controlled intake of carbohydrates, protein, and fat under the guidance of nutritionists effectively reduced excessive fat and protein intake, achieving a favorable clinical intervention effect (32). (2) Aerobic exercise increased the number, volume, and enzyme activity of mitochondria, promoting fat decomposition and fatty acid oxidation, while reducing protein loss and increasing its synthesis (33).

In terms of glycolipid metabolism, our results showed that after intervention, TC and LDL-C decreased while TG and HDL-C improved in both groups, with more significant improvements in the study group. Similarly, FPG, FINS, C-P, and HOMA-IR levels decreased, with lower values in the study group. These findings suggest that the combined approach can improve glycolipid metabolism. The underlying mechanisms involve: (1) low-carbohydrate diets stabilize postprandial blood sugar, reduce the rate of blood sugar change, and increase insulin sensitivity by decreasing carbohydrate intake and insulin requirement (34). (2) Aerobic exercise improves glucose tolerance, reduces insulin secretion, and enhances lipid metabolism capacity, promoting the reduction of LDL-C, the increase of HDL-C, and the decomposition and transformation of TG (35).

Furthermore, our study also indicated that the combined approach can improve negative emotions and enhance the quality of life in children and adolescents with obesity. The reduction in SAS and SDS scores and the increase in social, psychological, physical, and emotional function scores in the study group, suggest that the dietary and exercise intervention positively impacts mental health. This may be due to the improved physical appearance, increased self-confidence, and better overall well-being resulting from weight loss and improved health status.

### 5.1 Strengths and limitations

Strengths of the study: (1) the low-carbohydrate and high-nutrient-density plan formulated in this study accounted for the unique growth and developmental needs of children. By setting the nutrient distribution ratio at 20–30% for carbohydrates (< 100 g/d), 40–50% for protein, and 20–30% for fat, and calculating the total caloric requirement for each patient based on their individual circumstances, we ensured a balanced and safe dietary intervention. This personalized approach is more likely to meet the specific nutritional requirements of each child and adolescent, which is crucial for their healthy growth and development during the intervention period. (2) Our study not only focused on traditional outcomes such as body composition changes by simultaneously evaluating the effects on mental health and glycolipid metabolism. This comprehensive evaluation provides a more holistic view of the intervention’s impact on this vulnerable population, allowing for a better understanding of how the combined approach affects multiple aspects of health.

Limitations of the study: (1) The sample size was relatively small, which may limit the generalizability of our results. A larger sample size would be needed to confirm that the observed effects are consistent across a more diverse group of children and adolescents with obesity. (2) The study duration was relatively short, and the long-term effects of the dietary and exercise intervention remain unclear. Extended follow-up studies are necessary to assess whether the improvements in body composition, glycolipid metabolism, and mental health are sustained over time and to identify any potential long-term adverse effects. (3) Adherence to the dietary and exercise intervention was not assessed in detail, which may have influenced the outcomes. Future studies should incorporate more comprehensive methods to monitor adherence, such as dietary recalls, food diaries, and wearable devices to track physical activity, to better understand the relationship between adherence and intervention effects. (4) This study was conducted in a specific population, and the results may not be generalized to populations with different cultural backgrounds or dietary habits. Different cultural norms and food preferences may affect the acceptance and effectiveness of the intervention, highlighting the need for further research in diverse populations.

### 5.2 Implications of the study

In the medical field, our study provides new evidence-based insights for the prevention and treatment of childhood and adolescent obesity. The combined approach of a low-carbohydrate and high-nutrient-density diet with aerobic exercise represents a potentially effective and safe intervention strategy that can simultaneously address multiple obesity-related health issues, such as body composition, glycolipid metabolism, and mental health. These findings may guide clinicians in developing more comprehensive and personalized treatment plans for young patients with obesity, thereby potentially improving treatment outcomes and reducing the risk of long-term complications.

From a societal perspective, childhood and adolescent obesity is a major public health concern with significant economic and social impacts. If confirmed by further research, our findings can contribute to reducing the prevalence of obesity and its associated health problems in this population. This, in turn, may decrease healthcare costs pertaining obesity-related diseases, such as diabetes, cardiovascular diseases, and mental health disorders. Additionally, improving the health and well-being of children and adolescents can positively affect educational attainment, social integration, and future productivity, ultimately benefiting society as a whole.

## 6 Conclusion

Our study suggests that a low-carbohydrate and high-nutrient-density diet combined with aerobic exercise can improve mental health and glycolipid metabolism in children and adolescents with obesity. However, due to the limitations of this study, including the relatively small sample size, short study duration, and lack of detailed adherence assessment, further research is required to confirm these findings and to explore the long-term effects and optimal implementation strategies of this combined approach.

## Data Availability

The datasets presented in this study can be found in online repositories. The names of the repository/repositories and accession number(s) can be found in the article/supplementary material.

## References

[1] Sommer A Twig G. The impact of childhood and adolescent obesity on cardiovascular risk in adulthood: a systematic review. Curr Diab Rep. (2018) 18:91. doi: 10.1007/s11892-018-1062-9, 30167798

[2] Hills AP Andersen LB Byrne NM. Physical activity and obesity in children. Br J Sports Med. (2011) 45:866–70. doi: 10.1136/bjsports-2011-090199, 21836171

[3] Pan XF Wang L Pan A. Epidemiology and determinants of obesity in China. Lancet Diabetes Endocrinol. (2021) 9:373–92. doi: 10.1016/S2213-8587(21)00045-0, 34022156

[4] Lister NB Baur LA Felix JF Hill AJ Marcus C Reinehr T . Child and adolescent obesity. Nat Rev Dis Primers. (2023) 9:24. doi: 10.1038/s41572-023-00435-4, 37202378

[5] Weihrauch-Blüher S Schwarz P Klusmann JH. Childhood obesity: increased risk for cardiometabolic disease and cancer in adulthood. Metabolism. (2019) 92:147–52. doi: 10.1016/j.metabol.2018.12.001, 30529454

[6] Simmonds M Llewellyn A Owen CG Woolacott N. Predicting adult obesity from childhood obesity: a systematic review and meta-analysis. Obes Rev. (2016) 17:95–107. doi: 10.1111/obr.12334, 26696565

[7] Dabas A Seth A. Prevention and Management of Childhood Obesity. Indian J Pediatr. (2018) 85:546–53. doi: 10.1007/s12098-018-2636-x, 29457204

[8] Godina-Flores NL Gutierrez-Gómez YY García-Botello M López-Cruz L Moreno-García CF Aceves-Martins M. Obesity and its association with mental health among Mexican children and adolescents: systematic review. Nutr Rev. (2023) 81:658–69. doi: 10.1093/nutrit/nuac083, 36164834 PMC10170326

[9] Kiss O Harkness A Müller-Oehring EM Nagata JM Baker FC. Associations between sleep, obesity, and mental health in adolescents: understanding sex-specific vulnerabilities. J Affect Disord. (2025) 391:119883. doi: 10.1016/j.jad.2025.119883, 40652980 PMC12632359

[10] Förster LJ Vogel M Stein R Hilbert A Breinker JL Böttcher M . Mental health in children and adolescents with overweight or obesity. BMC Public Health. (2023) 23:135. doi: 10.1186/s12889-023-15032-z, 36658514 PMC9849834

[11] Seals DR Nagy EE Moreau KL. Aerobic exercise training and vascular function with ageing in healthy men and women. J Physiol. (2019) 597:4901–14. doi: 10.1113/JP277764, 31077372 PMC6773490

[12] Hashida R Kawaguchi T Bekki M Omoto M Matsuse H Nago T . Aerobic vs. resistance exercise in non-alcoholic fatty liver disease: a systematic review. J Hepatol. (2017) 66:142–52. doi: 10.1016/j.jhep.2016.08.023, 27639843

[13] Zhang H Liang JL Wu QY Li JX Liu Y Wu LW . Swimming suppresses cognitive decline of HFD-induced obese mice through reversing hippocampal inflammation, insulin resistance, and BDNF level. Nutrients. (2022) 14:432. doi: 10.3390/nu14122432, 35745162 PMC9228449

[14] Song-Hua Y Lu W Kuan Z. Effects of different movement modes on plantar pressure distribution patterns in obese and non-obese Chinese children. Gait Posture. (2017) 57:28–34. doi: 10.1016/j.gaitpost.2017.05.001, 28551468

[15] Tang Z Ming Y Wu M Jing J Xu S Li H . Effects of caloric restriction and rope-skipping exercise on cardiometabolic health: a pilot randomized controlled trial in young adults. Nutrients. (2021) 13:222. doi: 10.3390/nu13093222, 34579097 PMC8467906

[16] Phomsoupha M Laffaye G. The science of badminton: game characteristics, anthropometry, physiology, visual fitness and biomechanics. Sports Med. (2015) 45:473–95. doi: 10.1007/s40279-014-0287-2, 25549780

[17] Tomiyama H. Routine stair climbing for vascular health. Hypertens Res. (2021) 44:1357–8. doi: 10.1038/s41440-021-00701-6, 34282308 PMC8287103

[18] Chao AM Quigley KM Wadden TA. Dietary interventions for obesity: clinical and mechanistic findings. J Clin Invest. (2021) 131:65. doi: 10.1172/JCI140065, 33393504 PMC7773341

[19] Ramírez-Manent JI Tomás-Gil P Martí-Lliteras P Coll Villalonga JL Martínez-Almoyna Rifá E López-González ÁA. Dietary intervention on overweight and obesity after confinement by COVID-19. Nutrients. (2023) 15:912. doi: 10.3390/nu15040912, 36839270 PMC9960430

[20] Wiechert M Holzapfel C. Nutrition concepts for the treatment of obesity in adults. Nutrients. (2021) 14:169. doi: 10.3390/nu14010169, 35011045 PMC8747374

[21] Liu C Meng Q Zu C Wei Y Su X Zhang Y . Dietary low- and high-quality carbohydrate intake and cognitive decline: a prospective cohort study in older adults. Clin Nutr. (2023) 42:1322–9. doi: 10.1016/j.clnu.2023.06.021, 37413810

[22] Rafiullah M Musambil M David SK. Effect of a very low-carbohydrate ketogenic diet vs recommended diets in patients with type 2 diabetes: a meta-analysis. Nutr Rev. (2022) 80:488–502. doi: 10.1093/nutrit/nuab040, 34338787

[23] Troesch B Biesalski HK Bos R Buskens E Calder PC Saris WH . Increased intake of foods with high nutrient density can help to break the intergenerational cycle of malnutrition and obesity. Nutrients. (2015) 7:6016–37. doi: 10.3390/nu7075266, 26197337 PMC4517043

[24] Papadopoulou SK Nikolaidis PT. Low-carbohydrate diet and human health. Nutrients. (2023) 15:2004. doi: 10.3390/nu15082004, 37111222 PMC10143153

[25] Neyman A Hannon TS. Low-carbohydrate diets in children and adolescents with or at risk for diabetes. Pediatrics. (2023) 152:755. doi: 10.1542/peds.2023-063755, 37718964

[26] Jafari-Maram S Daneshzad E Brett NR Bellissimo N Azadbakht L. Association of low-carbohydrate diet score with overweight, obesity and cardiovascular disease risk factors: a cross-sectional study in Iranian women. J Cardiovasc Thorac Res. (2019) 11:216–23. doi: 10.15171/jcvtr.2019.36, 31579462 PMC6759613

[27] Tiksnadi BB Triani N Fihaya FY Turu’Allo IJ Iskandar S Putri DAE. Validation of hospital anxiety and depression scale in an Indonesian population: a scale adaptation study. Fam Med Community Health. (2023) 11:e001775. doi: 10.1136/fmch-2022-001775, 37277187 PMC10255233

[28] Grassi M Nucera A. Dimensionality and summary measures of the SF-36 v1.6: comparison of scale- and item-based approach across ECRHS II adults population. Value Health. (2010) 13:469–78. doi: 10.1111/j.1524-4733.2009.00684.x, 20088893

[29] Jebeile H Kelly AS O'Malley G Baur LA. Obesity in children and adolescents: epidemiology, causes, assessment, and management. Lancet Diabetes Endocrinol. (2022) 10:351–65. doi: 10.1016/s2213-8587(22)00047-x, 35248172 PMC9831747

[30] López-Gil JF García-Hermoso A Sotos-Prieto M Cavero-Redondo I Martínez-Vizcaíno V Kales SN. Mediterranean diet-based interventions to improve anthropometric and obesity indicators in children and adolescents: a systematic review with Meta-analysis of randomized controlled trials. Adv Nutr. (2023) 14:858–69. doi: 10.1016/j.advnut.2023.04.011, 37127186 PMC10334150

[31] Shai I Schwarzfuchs D Henkin Y Shahar DR Witkow S Greenberg I . Weight loss with a low-carbohydrate, Mediterranean, or low-fat diet. N Engl J Med. (2008) 359:229–41. doi: 10.1056/NEJMoa0708681, 18635428

[32] Foster GD Wyatt HR Hill JO McGuckin BG Brill C Mohammed BS . A randomized trial of a low-carbohydrate diet for obesity. N Engl J Med. (2003) 348:2082–90. doi: 10.1056/NEJMoa022207, 12761365

[33] Porflitt-Rodríguez M Guzmán-Arriagada V Sandoval-Valderrama R Tam CS Pavicic F Ehrenfeld P . Effects of aerobic exercise on fibroblast growth factor 21 in overweight and obesity. A systematic review. Metabolism. (2022) 129:155137. doi: 10.1016/j.metabol.2022.155137, 35038422

[34] Yang Z Mi J Wang Y Xue L Liu J Fan M . Effects of low-carbohydrate diet and ketogenic diet on glucose and lipid metabolism in type 2 diabetic mice. Nutrition. (2021) 89:111230. doi: 10.1016/j.nut.2021.111230, 33838492

[35] Geng L Liao B Jin L Huang Z Triggle CR Ding H . Exercise alleviates obesity-induced metabolic dysfunction via enhancing FGF21 sensitivity in adipose tissues. Cell Rep. (2019) 26:2738–52.e4. doi: 10.1016/j.celrep.2019.02.014, 30840894

